# Supplementary material for: Twelve-month specific IgG response to SARS-CoV-2 receptor-binding domain among COVID-19 convalescent plasma donors in Wuhan
Source: Nat Commun. 2021 Jul 6;12:4144. doi: 10.1038/s41467-021-24230-5 (PMC8260809; doi:10.1038/s41467-021-24230-5)
Supplement: Supplementary file 1 — Supplementary Information [file 41467_2021_24230_MOESM1_ESM.pdf]

Supplementary Materials for

Twelve-month specific IgG response to SARS-CoV-2 receptor-binding domain among COVID-19 convalescent plasma donors in Wuhan

Cesheng Li[1,7], Ding Yu[2,3,7], Xiao Wu[1], Hong Liang[2,3], Zhijun Zhou[1], Yong Xie[1,3], Taojing Li[3], Junzheng Wu[2], Fengping Lu[1], Lu Feng[1], Min Mao[1], Lianzhen Lin[1], Huanhuan Guo[1], Shenglan Yue[1], Feifei Wang[1], Yan Peng[1], Yong Hu[1], Zejun Wang[4], Jianhong Yu[1], Yong Zhang[3], Jia Lu[4], Haoran Ning[1], Huichuan Yang[5], Daoxing Fu[3], Yanlin He[1,3], Dongbo Zhou[3], Tao Du[3], Kai Duan[4], Demei Dong[3], Kun Deng[1], Xia Zou[1], Ya Zhang[1], Rong Zhou[3], Yang Gao[3], Xinxin Zhang[6,8] & Xiaoming Yang[5,8]

[1]Sinopharm Wuhan Plasma-derived Biotherapies Co., Ltd, 430207 Wuhan, China.

[2]Chengdu Rongsheng Pharmaceuticals Co., Ltd, 610041 Chengdu, China.

[3]Beijing Tiantan Biological Products Co., Ltd, 100024 Beijing, China.

[4] Wuhan Institute of Biological Products Co. Ltd., 430207 Wuhan, China.

[5] China National Biotec Group Company Limited, 100029 Beijing, China.

[6]Research Laboratory of Clinical Virology, Ruijin Hospital and Ruijin Hospital North, National Research Center for Translational Medicine, Shanghai Jiao Tong University of Medicine, 200025 Shanghai, China.

[7]Equal contributing authors
[8]Jointly supervising authors

Supplemental Table 1 Percentage changes of RBD-IgG titers distribution of COVID-19 convalescent plasma donors at early, middle, and late stages following diagnosis.

	**Percentage (%)**		
	**1-2 months**	**6-7 months**	**11-12 months**
1:2560	11.28	1.14	0.90
1:1280	17.95	4.55	1.65
1:640	26.67	9.47	6.16
1:320	16.67	26.14	18.47
1:160	15.64	31.06	29.73
1:80	6.41	17.05	24.32
<1:80	5.38	10.61	18.77

Supplemental Table 2 Percentage changes of RBD-IgG titers in plasma donors with low, moderate, or high titers after a long period of time. Negative, titers less than 1:80. High (high titers), titers of 1:1280 and ≥ 1:2560. Moderate (moderate titers), titers of 1:320-1:640. Low (low titers), titers of 1:80-1:160.

		**% at 10-11 months**			
		**High**	**Moderate**	**Low**	**Negative**
Initial	High (n=70)	8.57	54.29	35.71	1.43
	Moderate (n=107)	1.87	24.30	60.75	13.08
	Low (n=60)	0.00	11.67	36.67	51.67
