# Supplementary material for: Twelve-month specific IgG response to SARS-CoV-2 receptor-binding domain among COVID-19 convalescent plasma donors in Wuhan
Source: Nat Commun. 2021 Jul 6;12:4144. doi: 10.1038/s41467-021-24230-5 (PMC8260809; doi:10.1038/s41467-021-24230-5)
Supplement: Supplementary file 3 — Reporting Summary [file 41467_2021_24230_MOESM3_ESM.pdf]

nature research

Corresponding author(s): Xiaoming Yang

Last updated by author(s): 2021-5-30

Reporting Summary

Nature Research wishes to improve the reproducibility of the work that we publish. This form provides structure for consistency and transparency in reporting. For further information on Nature Research policies, see our Editorial Policies and the Editorial Policy Checklist.

Statistics

For all statistical analyses, confirm that the following items are present in the figure legend, table legend, main text, or Methods section.

n/a	Confirmed	
☐	☒	The exact sample size (n) for each experimental group/condition, given as a discrete number and unit of measurement
☐	☒	A statement on whether measurements were taken from distinct samples or whether the same sample was measured repeatedly
☐	☒	The statistical test(s) used AND whether they are one- or two-sided<br>*Only common tests should be described solely by name; describe more complex techniques in the Methods section.*
☒	☐	A description of all covariates tested
☒	☐	A description of any assumptions or corrections, such as tests of normality and adjustment for multiple comparisons
☐	☒	A full description of the statistical parameters including central tendency (e.g. means) or other basic estimates (e.g. regression coefficient) AND variation (e.g. standard deviation) or associated estimates of uncertainty (e.g. confidence intervals)
☐	☒	For null hypothesis testing, the test statistic (e.g. F, t, r) with confidence intervals, effect sizes, degrees of freedom and P value noted<br>*Give P values as exact values whenever suitable.*
☒	☐	For Bayesian analysis, information on the choice of priors and Markov chain Monte Carlo settings
☒	☐	For hierarchical and complex designs, identification of the appropriate level for tests and full reporting of outcomes
☒	☐	Estimates of effect sizes (e.g. Cohen's d, Pearson's r), indicating how they were calculated

Our web collection on statistics for biologists contains articles on many of the points above.

Software and code

Policy information about availability of computer code

Data collection	Only Excel was used for data collection.
Data analysis	GraphPad Prism 9.0 ; SoftMax5.2

For manuscripts utilizing custom algorithms or software that are central to the research but not yet described in published literature, software must be made available to editors and reviewers. We strongly encourage code deposition in a community repository (e.g. GitHub). See the Nature Research guidelines for submitting code & software for further information.

Data

Policy information about availability of data

All manuscripts must include a data availability statement. This statement should provide the following information, where applicable:

- Accession codes, unique identifiers, or web links for publicly available datasets
- A list of figures that have associated raw data
- A description of any restrictions on data availability

The authors declare that the data supporting the findings of this study are available within the paper and its supplementary and Source files. Other data that support the findings of this study are available from the corresponding author upon reasonable request.

Field-specific reporting

Life sciences study design

All studies must disclose on these points even when the disclosure is negative.

Sample size	The sample size was not determined by statistical methods. This study lasted for 12 months and used a total of 1782 plasma samples. The number of plasma samples collected each month was kept at least in double digits. We divided the 12-month study cycle into three periods: the early stage (1st and 2nd months), middle stage (6th and 7th months), and later stage (11th and 12th months) following diagnosis. The plasma samples used in each period were 390, 264 and 666, respectively, reaching the order of three digits. Therefore, we also believe that the plasma sample size included in this study is sufficient.
Data exclusions	Among 869 COVID-19 convalescent plasma donors, blood type data were contained in 834 individuals. Therefore, 35 plasma samples were excluded from blood type correlation analysis. No other data were excluded from any analysis.
Replication	Samples were assayed with ELISA and PRNT. Basically the sample was test once, if the sample result is out of ranged, it will be diluted properly to test again.
Randomization	n/a . We aimed to monitor the changes of antibody in COVID-19 patients where randomization was not applicable to this study.
Blinding	n/a. Since this study is an observational research but not an RCT. Participates involved in this study did not receive any drugs. Therefore, blinding was not applicable.

Reporting for specific materials, systems and methods

We require information from authors about some types of materials, experimental systems and methods used in many studies. Here, indicate whether each material, system or method listed is relevant to your study. If you are not sure if a list item applies to your research, read the appropriate section before selecting a response.

Materials & experimental systems

n/a	Involved in the study	
☐	☒	Antibodies
☒	☐	Eukaryotic cell lines
☒	☐	Palaeontology and archaeology
☒	☐	Animals and other organisms
☐	☒	Human research participants
☒	☐	Clinical data
☒	☐	Dual use research of concern

Methods

n/a	Involved in the study	
☒	☐	ChIP-seq
☒	☐	Flow cytometry
☒	☐	MRI-based neuroimaging

Antibodies

Antibodies used	An CE-marked Wantai SARS-CoV-2 IgG ELISA (Quantitative) kit (catalog number: WS-1396) from Beijing WanTai Biological Pharmacy Enterprise Co., Ltd. (Beijing, China) was used to test the titer of RBD-IgG in COVID-19 convalescent plasma samples. No other commercial antibodies were used.
Validation	n/a. COVID-19 convalescent plasma (assigned as 1:320, lot number: 2020021702, prepared by Sinopharm Wuhan Plasma-derived Biotherapies Co., Ltd.) was used as the reference standard. The target titer was obtained by 10 tests with WANTAI SARS-CoV-2 IgG kit. The titer of the reference standard is expressed as dilution and defined by the largest dilution when optical density (OD) value was higher than cut-off value of the kit. After validation of the linearity, range, and precision, the quantitative mothed was used to test samples

Human research participants

Policy information about studies involving human research participants

Population characteristics	The 869 COVID-19 convalescent plasma donors are all between 15 and 55 years old, and among all the donors, 478 (55%) are male and 391 (45%) are female. Among them, the blood type data of 834 individuals were collected, and the proportions of plasma Rh-positive blood types of A, B, O, and AB were 34.89%, 27.46%, 29.38%, and 8.27%, respectively.
Recruitment	From February 1, 2020 to January 10, 2021, 869 COVID-19 convalescent plasma donors in Wuhan, China were recruited, and 1,782 convalescent plasma samples were collected. All donors met the criteria for release of isolation and discharge from the hospital according to the "Diagnosis and Treatment Protocol for Novel Coronavirus Pneumonia (Trial Version 4 and subsequent versions)", released by the National Health Commission & State Administration of Traditional Chinese Medicine. Those who meet the criteria below could be discharged:<br>1) Body temperature was back to normal for more than three days;<br>2) Respiratory symptoms were obviously improved;<br>3) Pulmonary imaging showed obvious absorption of inflammation;<br>4) Nuclei acid tests were consecutively negative twice for respiratory tract samples such as sputum and nasopharyngeal swabs (with sampling interval being at least 24 hours).<br><br>biases: All the participates in this study were willing to donate plasma samples. Patients with severe symptoms were not suitable to donate plasma. Antibody changes of the patients with severe diseases were not fully assessed in this study.
Ethics oversight	The full name is Ethics Committee of Tiantan Biological R&D Center.<br>We have added the information in the manuscript. Please see "Ethics " section.

Note that full information on the approval of the study protocol must also be provided in the manuscript.
